# Rare Case of Grade 3 Undifferentiated Pleomorphic Sarcoma in Left Atrium, Mitral Valve, and Papillary Muscle

**DOI:** 10.3390/jcm14093053

**Published:** 2025-04-28

**Authors:** Silvia Preda, Kishore K. Gangangari, Robert Tiganasu, Andreea Liciu, Claudia Nica, Alexandra Voicu, Vlad Ichim, Horatiu Moldovan

**Affiliations:** 1 Faculty of Medicine, “Carol Davila” University of Medicine and Pharmacy, 050474 Bucharest, Romania; silvia-mihaela.pieleanu@drd.umfcd.ro (S.P.); tiganasu.robert@yahoo.com (R.T.); liciu.andreea@gmail.com (A.L.); bianca.nica@yahoo.com (C.N.); horatiu.moldovan@umfcd.ro (H.M.); 2 Department of Cardiovascular Surgery, Clinical Emergency Hospital Bucharest, 014461 Bucharest, Romania; 3 Department of Cardiovascular Anesthesia and Intensive Care, Clinical Emergency Hospital Bucharest, 014461 Bucharest, Romania; alexandra.voicu@rez.umfcd.ro; 4 Academy of Romanian Scientists, 050044 Bucharest, Romania

**Keywords:** intracardiac tumor, undifferentiated pleomorphic sarcoma, rapid deep cooling, cerebral embolism

## Abstract

**Background**: Primary intracardiac tumors may be diagnosed incidentally, sometimes in the case of complications. **Case Report**: This case report presents a 64-year-old woman who was admitted to the emergency department with cardiac complications, including heart palpitations and shortness of breath. Initial investigations revealed the presence of ground glass opacity in the left lung and significant mediastinal adenopathy. Transthoracic echocardiography (TTE) indicated severe mitral stenosis caused by a mass attached to the mitral valve, and the transesophageal echocardiography (TEE) confirmed the presence of a tumor, raising concerns about a myxoma with a high risk of embolism. The patient experienced transitory neurological dysfunction, and subsequent imaging uncovered a thrombus occluding the left internal carotid artery. An emergency surgical procedure was performed, including extracorporeal circulation and rapid deep cooling, to facilitate safe mass excision and carotid embolectomy. Histopathological analysis of the extracted tissue revealed undifferentiated pleomorphic sarcoma (FNCLCC Grade 3). Following the surgery, the patient needed extended mechanical ventilation and subsequently underwent a tracheostomy because of her ongoing respiratory support requirements. **Conclusions**: Despite the complexity of the surgical intervention, the prognosis remained poor due to the aggressive nature of the tumor and neurologic complications. This case underscores the rarity of primary cardiac sarcomas, the challenges in diagnosis, and the need for prompt surgical intervention to mitigate risks associated with embolic events.

## 1. Introduction

Intracardiac tumors are rare but can have dreadful complications. Unfortunately, malignant tumors have a poor prognosis with a survival rate of around 50% at 1 year with surgical and oncological treatment. It also depends on the biological status and comorbidities of the patient, type of tumor, its localization, and the possibility of total excision, and last but not least, the surgical procedures and perioperative complications [1]. Although they are extremely rare, cardiac tumors play a significant role in the field of cardio-oncology, where accurate diagnosis and management are crucial. These tumors encompass a wide variety of lesions and masses, which can be classified as either neoplastic or non-neoplastic. Neoplastic lesions are further divided into primary and secondary tumors, with secondary tumors indicating metastasis to the heart. Up to 90% of primary cardiac tumors (PCTs) are benign and can arise from the pericardium or myocardium. The incidence of clinically diagnosed PCTs is estimated to be about 1380 per 100 million individuals. In contrast, secondary cardiac tumors are 22 to 132 times more prevalent and are inherently malignant [1,2,3,4]. Classifying these lesions as benign or malignant is a key factor in determining prognosis; however, any cardiac tumor, regardless of its benign histology, can lead to significant hemodynamic or arrhythmic issues depending on its size and position within the heart [5].

In the last ten years, there has been a marked increase in the incidence of PCTs. This rise is partly due to advancements in imaging techniques, making multimodal imaging more accessible and widely used. Such imaging approaches are often essential for diagnosing PCTs. Important diagnostic elements before an open biopsy may include the mass’s location, imaging features, and patient age at presentation [1,6].

## 2. Case Report

A 64-year-old woman was admitted into the emergency ward following cardiac complications, including heart palpitations and shortness of breath, where the patient was treated conservatively. The mental state of the patient and neurological functions of the patient were normal when admitted.

**Figure 1:**
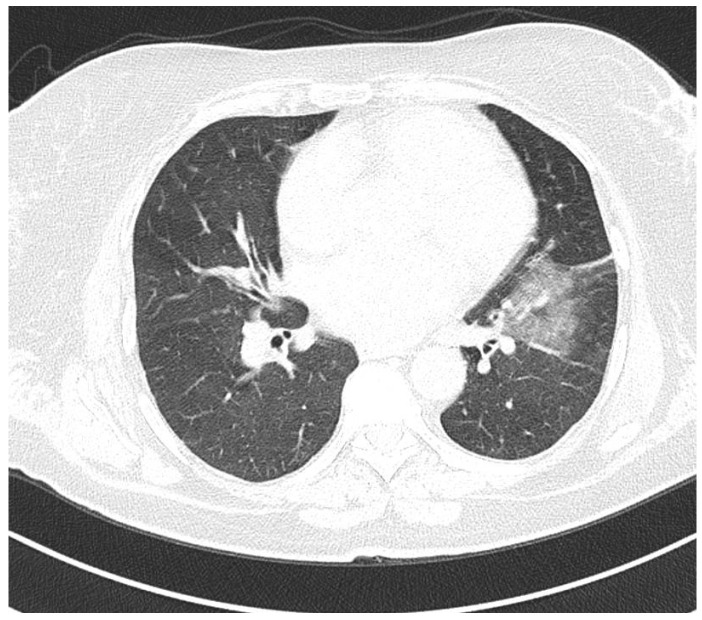
CT images revealing a ground glass opacity, measuring 41/44 mm at the level of medial and anterior segments of the lower lobe, was associated with cylindrical projections inside.

Initial chest computed tomography (CT) with contrast revealed a ground glass opacity (GGO), measuring 41 × 44 mm and located at the level of medial and anterior basal segments of the lower left lobe, that was associated with cylindrical projections inside (Figure 1). A fibrous band was observed adjacent to the GGO indicating mitral valve chordae. Additionally, 13 × 15 mm mediastinal adenopathy was observed in the right paratracheal lymph nodes. The tracheo-bronchial tree was free, and the thoracic artery was permeable with normal caliber. Most importantly, the pulmonary arteries were of normal caliber and were homogeneously opaque without intraluminal thrombi.

Transthoracic echocardiography showed that the left ventricle was not dilated, not hypertrophied, without segmental kinetic disorder, and 55–60% preserved LVEF (visual estimation). Diastolic dysfunction with increased filling pressure was observed with severely dilated AS. A voluminous mass was observed at the level of posterior mitral valve. Separately, a dilated right ventricle (RV) with systolic dysfunction (low RV fractional area change) and a moderate–severe tricuspid regurgitation with systolic dysfunction were observed.

**Figure 2:**
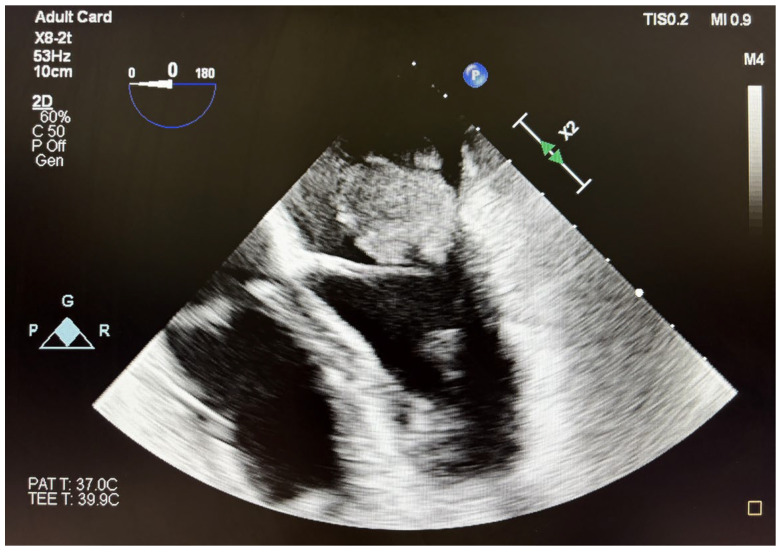
Preoperative TEE images of the intracardiac mass with a maximum diameter of 3 cm, attached to the P2/P3 scallop of the mitral valve.

A transesophageal echocardiography to characterize the formation attached to the mitral valve revealed a tumor attached to the poster leaflet at the level of P1/P2 with the longest dimension of 2.8 × 3 cm (7 cm^2^ area) causing severe mitral stenosis and mild regurgitation (Figure 2). These results were highly suggestive of a myxoma, with increased risk of embolism. An additional formation attached to the postero-medial papillary muscle with similar echogenicity was also observed, as opposed to the usual presentation of the myxomas.

Coronary angiography with the right radial approach had no procedural complications and showed permeable epicardial coronary arteries without lesions.

**Figure 3:**
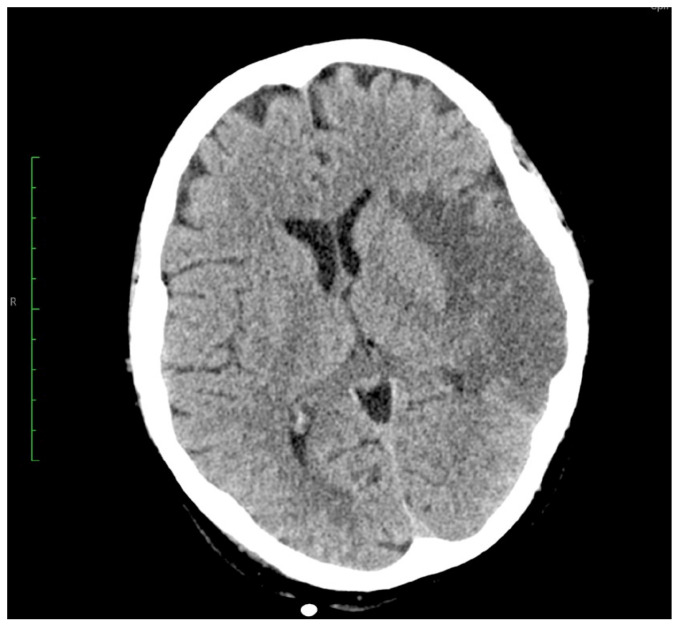
CT images of the head axial section showing hyperdense left M2 with hypodense lesion with ischemic appearance.

**Figure 4:**
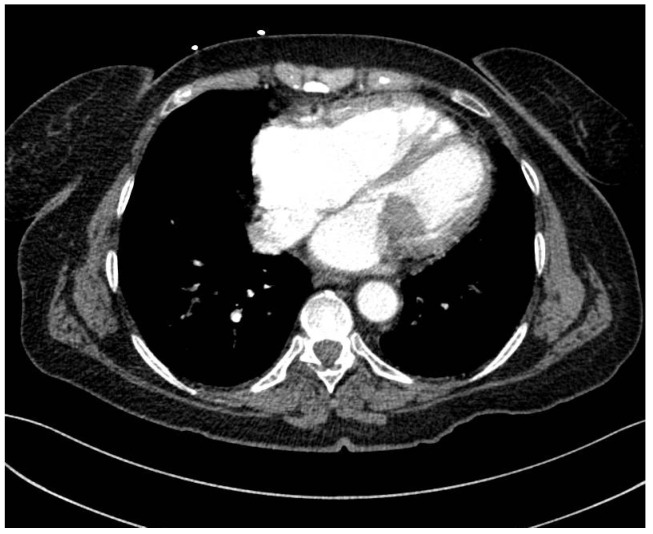
CT images of the thorax axial section with 3.6/2.6 cm hypodense nodule at the center of the mitral valve.

**Figure 5:**
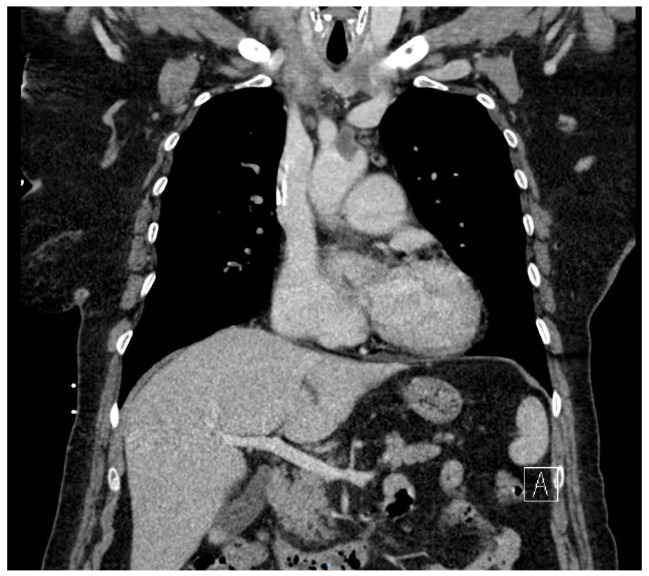
CT images of the thoracic coronal section showing the extension of the nodule into the aortic valve and embolus in the aortic arch.

The patient experienced transitory neurological dysfunctions, and a confirmatory contrast CT was performed. Cranial CT showed a hyperdense left M2 with hypodense lesion with ischemic appearance (Figure 3). The thoracic CT scan showed a 3.6 × 2.6 cm hypodense nodule at the center of mitral valve, with extension toward the aortic valve (Figure 4). Furthermore, a new occlusion in the left internal carotid artery was observed due to a thrombus (at least 5 cm) starting from its emergence from the aorta (Figure 5). Pulmonary arteries were permeable. We used a GE Healthcare Japan Corporation, Tokyo, Japan, model Optima CT660, year of fabrication 2015 to perform the emergency scan.

Given the acute nature of diagnosis of a potential myxoma with high risk for embolism, an immediate emergency surgery was scheduled to remove the mass. Prior to the surgery, the patient experienced multiple transitory cerebral attacks, as well as an ischemic attack with the right hemiplegia and aphasia. 

**Figure 6:**
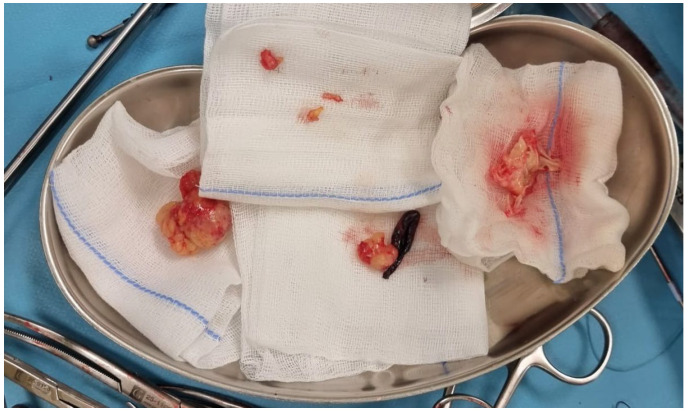
Intraoperative view of the carotid embolus extracted from the aortic arch.

**Figure 7:**
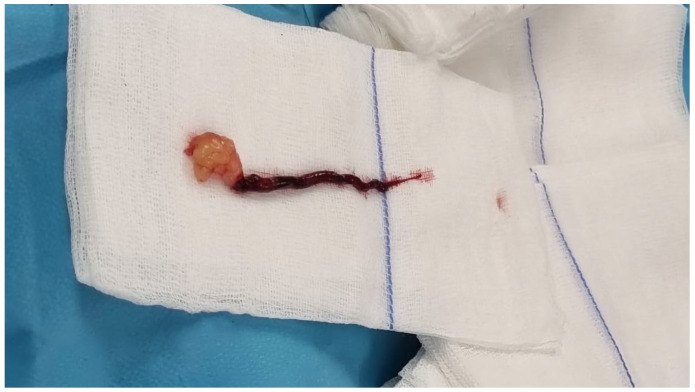
Intraoperative view of the cardiac tumor with the embolus and the whole mitral subvalvular apparatus resected.

Considering the recent symptomatology of stroke of <12 h and the proof of left carotid embolus, we decided to intervene immediately, first to establish the normal cerebral perfusion and then to remove the intracardiac mass. The embolus was situated in the aortic arch, appeared fragile on the ultrasound images, and posed a significant risk of further embolization to the carotid artery or other cerebral vessels. Therefore, we believed that conducting an open arch procedure, which would allow us to maintain complete control over the cerebral vessels while minimizing additional manipulation, was the safest approach. To excise the mass safely, we initiated extracorporeal circulation through central cannulation and started rapid deep cooling to 18 °C. Meanwhile, we made a laterocervical incision to have the left common carotid artery exposed and clamped at the time of circulatory arrest to prevent distal embolization to the brain. When we reached 18 °C, we stopped the extracorporeal circulation and incised the aortic arch to better visualize the origin of cerebral vessels and to safely extract the embolus with the left carotid clamped. The embolus was floating in the aortic arch and occluded the carotid artery (Figure 6). The embolus was easily extracted, and the carotid artery was flushed to remove any small debris left. We first closed the aortic arch, restarted the extracorporeal circulation, and warmed the patient. Simultaneously, we de-aired and closed the left carotid artery. NIRS monitoring of the brain was normal throughout the procedure. While the patient was warmed, we entered the left atrium through the interatrial groove and visualized the tumor that included P2 and had a dynamic obstruction of the mitral orifice. We excised the posterior and anterior cusps and took into consideration the preserving of parts of the mitral subvalvular apparatus, but we also observed smaller tumors on the posteromedial papillary muscle and chordae, so we decided to take out the whole subvalvular apparatus as well (Figure 7). After a prior discussion with the family, we implanted a 29 mm biological valve given her neurological status and prognosis. After the mitral prosthesis implantation and reaching 36.5 °C, we finished the procedure in a standard fashion. TEE showed moderate LV dysfunction and normal mitral prosthesis with no leak.

The intraoperative details of the procedure were as follows: a total 125 min of extracorporeal circulation, with an aortic clamping time of 108 min and 18 min of circulatory arrest needed for the embolectomy of the aortic arch. The embolus extracted from the arch/left common carotid artery had a length of 10 cm and the tumor was oval-shaped with a maximum diameter of 4 cm.

**Figure 8:**
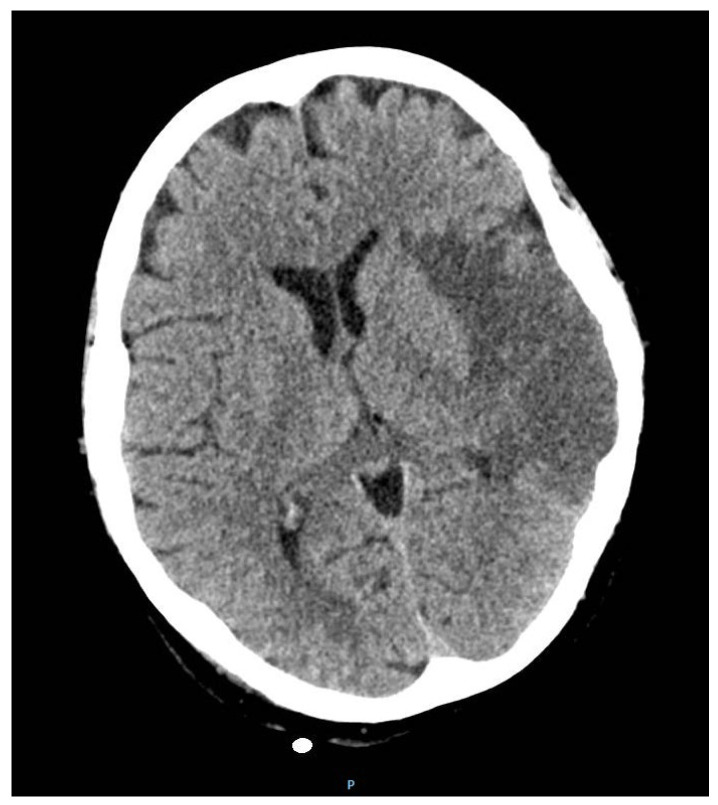
Cerebral CT axial images showing the stationary ischemic lesions from preop.

To follow up on cerebral attacks, a cranial CT was performed three days post-op (Figure 8) which showed hyperdense left M2 with hypodense lesion with ischemic appearance. However, no ischemic lesions were observed in the right cerebral or cerebellar hemisphere, and no intracranial blood accumulation were observed.

**Figure 9:**
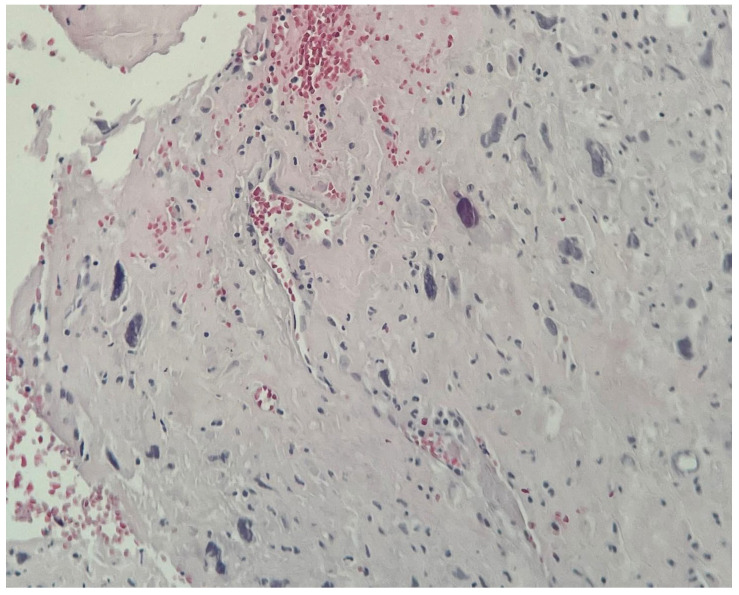
Microscopic image of the tissue fragments from the mitral valve.

**Figure 10:**
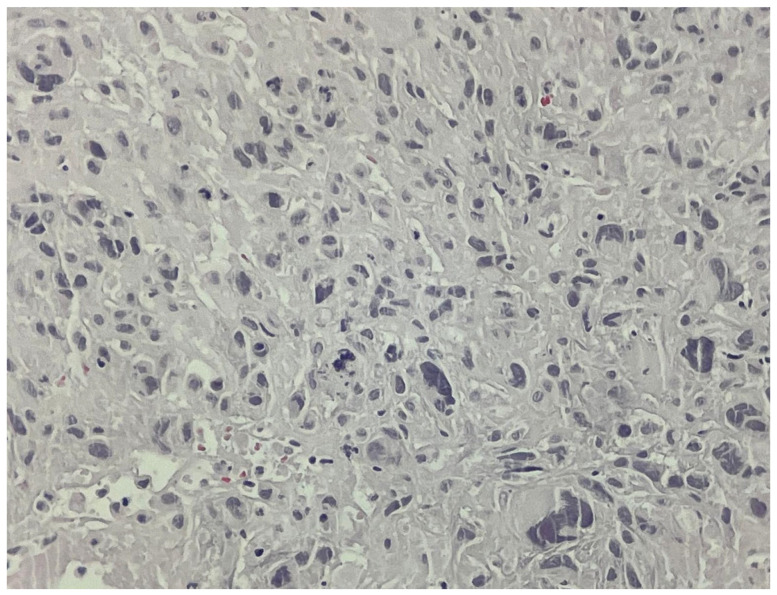
Microscopic image of the tissue fragments from the papillary muscle.

Three separate tissue samples (left atrium, mitral valve, and papillary muscle) were extracted, and their histopathology was investigated by immunohistochemistry (IHC). All samples tested positive for tumoral aspects, and the IHC supported the diagnosis of undifferentiated pleomorphic sarcoma (FNCLCC Grade 3). Figure 9 and Figure 10 show malignant tumor proliferation, characterized by spindle-shaped epithelioid and stellate cells, with marked nuclear pleomorphism, as well as frequent bizarre giant cells that were occasionally multinucleated. Mitotic activity is noted at 16 mitoses per 10 high-power fields (HPF), with the presence of tumor necrosis and extensive coagulative necrosis. Vascular invasion is evident (LV1), and tumor characteristics are observed at the margins of the fragments.

Immunohistochemical Tests: Destin—positive (focal), CD31—positive (focal), CD34—positive (focal), CD68—positive (focal), WT1—negative, calretinin—positive (focal), S100—positive (focal), Myogenin—negative, Ki67 at 40%.

The histopathological features and immunohistochemical tests support a diagnosis of undifferentiated pleomorphic sarcoma, FNCLCC grade 3.

## 3. Outcome and Follow-Up

No complications were noted during the surgery. However, the patient needed intubation following the surgery and was placed on mechanical ventilation in the ICU. The patient needed prolonged ventilation and, in order to wean her from the mechanical ventilation, she needed a tracheostomy. Her pre-op neurological status persisted but no other new deficits were observed. The needed rehabilitation is expected to be mostly neurological in nature following the ischemic attack. The prognosis was poor considering her status and type of tumor. The patient had a prolonged hospital stay and was transferred to a rehabilitation center where she recovered partially. She did not receive chemotherapy immediately and we know that she was in good clinical status almost 1 year after surgery because she underwent her cardiologic reevaluation at another center. Unfortunately, we do not know if she finally started chemotherapy or if she is still alive. We could not reach her on the phone.

## 4. Discussion

While sarcomas originating in the heart are rare, they have been previously reported in the literature, including some distinct types, such as spindle cell sarcomas [7,8,9,10]. Due to their rarity, they are often differentially diagnosed as benign tumors or myxomas. Correlatively, their detection rate is low and are often diagnosed in much later stages, potentially metastasizing and/or leading to embolisms, resulting in a much poorer prognosis rate. For accurate diagnosis, multi-modal imaging and diagnostic techniques, potential use of genetic testing to identify the targets in primary sarcomas [11], along with efforts towards increasing awareness of rare cancers in heart diseases are needed for a potential early diagnosis and betterment of prognosis.

From the outset, we suspected the presence of a cardiac neoplasm, given the multiple intracardiac tumors, the echographic characteristics, and the infiltrative nature observed during the surgical procedure. The intervention successfully excised as much tissue as feasible under safe conditions, with the definitive diagnosis established through pathological analysis.

CT is a quick and highly available imaging technique, the high temporal resolution of new CT scanners allows a low-dose, fast, and highly defined imaging acquisition; moreover, an infiltrative growth pattern can be suggested by CT images and, when possible, confirmed by MR imaging.

MR is renowned to be the best imaging modality due to the spontaneous contrast between tissue components; therefore, it is the ideal tool in such a pathology. However, MR examinations are quite long and need patients’ collaboration, not to mention that their availability is lower than CT.

In some case reports, patients with undifferentiated pleomorphic cardiac sarcomas have experienced survival durations of only a few weeks without oncologic treatment. This highlights the aggressive nature of the disease and the critical need for effective management strategies to prolong survival [12]. Cardiac MRI offers advantages over echocardiography, including superior spatial resolution, a wider field of view, and detailed tissue characterization, which helps identify cardiac tumors that may not need further intervention. However, it may not be specific enough to differentiate between the left atrial undifferentiated pleomorphic sarcoma and other tumors, such as myxomas. Full-body positron emission tomography (PET) is useful for evaluating active metabolic lesions and identifying metastatic foci in patients. This imaging technique helps to detect areas of increased metabolic activity that may indicate the presence of cancer or other abnormalities, aiding in the overall assessment and management of the disease [13].

The surgical procedure is complex, and we deployed novel interventional techniques to remove the tumors. Rapid deep cooling is a safe technique to employ procedures involving the aortic arch and brain, avoiding prolonged extracorporeal circulation time akin to normothermia/moderate hypothermia [14]. This newly improved technique of cooling to deep hypothermia avoids many of the complications due to a long time of extracorporeal circulation needed for slow cooling and rewarming [15]. In case of a suspected malignant tumor, radical surgery with predictable, favorable outcomes should be preferred. Rapid carotid occlusion embolectomy should be performed, if possible, within the first four hours to lower the risk of neurological dysfunction.

## Data Availability

Data available upon request.
